# Analysis of Complete Mitochondrial Genome of *Bohadschia argus* (Jaeger, 1833) (Aspidochirotida, Holothuriidae)

**DOI:** 10.3390/ani12111437

**Published:** 2022-06-02

**Authors:** Bo Ma, Zhuobo Li, Ying Lv, Zixuan E, Jianxiang Fang, Chunhua Ren, Peng Luo, Chaoqun Hu

**Affiliations:** 1 CAS Key Laboratory of Tropical Marine Bioresources and Ecology (LMB), South China Sea Institute of Oceanology, Chinese Academy of Sciences, Guangzhou 510301, China; mabo20@mails.ucas.ac.cn (B.M.); lizhuobo19@mails.ucas.ac.cn (Z.L.); ezixuan20@mails.ucas.ac.cn (Z.E.); fangjianxiang18@mails.ucas.ac.cn (J.F.); renchunhua@scsio.ac.cn (C.R.); hucq@scsio.ac.cn (C.H.); 2 Southern Marine Science and Engineering Guangdong Laboratory (Guangzhou), Guangzhou 510301, China; 3 University of Chinese Academy of Sciences, Beijing 100049, China; 4 Guangxi Key Laboratory of Beibu Gulf Marine Biodiversity Conservation, Beibu Gulf University, Qinzhou 535011, China; lvying0211@163.com

**Keywords:** Holothuroidea, Aspidochirotide, *Bohadschia*, mitochondrial genome, phylogeny

## Abstract

**Simple Summary:**

Mitochondrial genomes are a type of specific genetic marker, characterized in most animals by a rapid mutation rate, high copy numbers and the absence of genetic recombination. They are widely applied in phylogenetics, population genetics and ecological research. *Bohadschia argus* is a sea cucumber with high economic value. Currently, the mitochondrial genomic data of *B. argus* are not available. In this study, next-generation sequencing was performed on the mitochondrial genome of *B. argus*. The gene arrangement order in the mitochondrial genome of *B. argus* was consistent with the echinoderm ground pattern. Finally, phylogenetic analysis using the 13 protein-coding genes of mitochondria confirmed the phylogenetic position of *B. argus*.

**Abstract:**

*Bohadschia argu* is a kind of sea cucumber with high economic value; it is the only undisputed species in the genus *Bohadschia*. In this study, the complete mitochondrial genome (mitogenome) of *B. argus* was acquired through high-throughput sequencing. The mitochondrial genome of *B. argus* was 15,656 bp in total length and contained a putative control region (CR) and 37 typical genes of animal mitochondrial genomes, including 13 protein-coding genes (PCGs), 2 ribosomal RNA genes (*rrnS* and *rrnL*) and 22 transfer RNA genes (tRNA). The sizes of the PCGs ranged from 168 bp to 1833 bp, and all PCGs except *nad6* were encoded on the heavy chain (H). Both *rrnS* and *rrnL* were also encoded on the H chain. Twenty-two tRNA genes had positive AT skew and GC skew. All tRNAs had a typical cloverleaf secondary structure except for *trnI*, in which an arm of dihydrouridine was missing. *B. argus* shared the same gene arrangement order (the echinoderm ground pattern) as other species in Aspidochirotida. Phylogenetic analysis clearly revealed that *B. argus* belongs as a member of the Holothuriidae, and it is closely related to members of *Actinopyga* and *Holothuria*.

## 1. Introduction

Holothuroids, commonly known as sea cucumbers, are an important group of benthic organisms, including abundant and diverse ecologically and economically important species [1]. Sea cucumbers belong to the phylum Echinodermata, which includes about 1400 species widely distributed throughout the Earth’s oceans [2,3]. Sea cucumbers in the genus *Bohadschia* have high commercial value as they are edible and hold medicinal value [4]; they belong to Holothuriidae, which contains the most diverse species in Aspidochirotida [5]. *B. argus* is loaf-like and has either light gray walls covered with gray ocellar spots or brown body walls covered with light beige ocellar spots [6]. Based on the above characteristics, the identification of *B. argus* is relatively easy.

Holothuroids are generally identified by their morphological characteristics, including tentacles, the presence or absence of tentacular retractor muscles, a respiratory tree and dermal ossicles [7]. However, similar to other animals, the phenotypic plasticity of sea cucumbers may be a result of interaction among environmental selective pressure, adaption, gene flow and genomic evolution [8,9,10,11]. In addition, morphological variations may be caused by methodological artifacts used to collect and preserve the specimens [12]. In these ways, phenotypic plasticity and methodological artifacts likely confuse the taxonomy of sea cucumbers. Mitochondria are highly abundant in animal tissues, which contain a mitochondrial genome independent of chromosomes. Due to the relatively small molecular sizes, maternal inheritance, low recombination rate and simple sequencing procedures of mitochondrial genomes, they are often used for analyzing phylogenetic relationships at multiple taxonomic levels [13]. Therefore, the mitochondrial genomes of animals have been extensively sequenced and studied. The complete mitochondrial genomes of 26 species of sea cucumber have been analyzed in detail, such as *Actinopyga lecanora*, *Thyonella gemmata*, *Stichopus chloronotus* and *Holothuria edulis* [14,15,16,17], but no mitochondrial genomes for *Bohadschia* species have been sequenced. As a representative species in *Bohadschia*, it is essential to obtain the whole mitochondrial genome sequence of *B. argus* to explore its taxonomic status. To date, the evolutionary position of *B. argus* has been analyzed only by using single genes. Based on previous phylogenetic analyses of multiple genes in sea cucumbers [14,15,16,17], it seems quite possible that carrying out phylogenetic analysis using 13 PCGs of the mitogenomes will more accurately define the evolutionary position of *B. argus*.

In that context, this study aimed to generate the complete sequence of the *B. argus* mitochondrial genome, to become the first publicly available mitochondrial genome in *Bohadschia*. Comparative genomics was carried out to characterize the mitochondrial genome of *B. argus*, and phylogenetic relationships among *B. argus* and other related sea cucumbers were analyzed based on the 13 PCGs in their mitochondrial genomes.

## 2. Materials and Methods

### 2.1. Sample Collection and DNA Extraction

There are no ethical implications for this study. By scuba diving, a specimen of *B. argus* was collected in Qionghai (N 19°25′, E 110°46′), Hainan Province, China, and stored in the Tropical Marine Biodiversity Collections of the South China Sea (TMBC) at the Chinese Academy of Sciences in Guangzhou, China (TMBC030973), which is managed by a contactable person (Xiping Lian, xplian@scsio.ac.cn). The sea cucumber specimen was identified as *B. argus* based on its morphological characteristics [6]. Total DNA was extracted from longitudinal muscle tissue using the Marine Tissue Genomic DNA Extraction Kit (TIANGEN BIOTECH, Beijing, China) according to the manufacturer’s instructions. Genomic DNA was stored at −20 °C.

### 2.2. Sequence Analysis and Gene Annotation

The complete mitochondrial genome of *B. argus* was sequenced using next-generation sequencing (BIOZERON Co., Ltd., Shanghai, China). Sequencing was carried out on the Illumina NovaSeq 6000 platform (2 × 150 bp paired-end reads were generated). An Illumina NovaSeq 6000 library with an insertion length of 450 bp was constructed. The raw data were processed using Trimmomatic v0.39 [18] as follows: remove the adapter sequences from the reads, cut off the bases containing non-AGCT at the 5′ end before trimming, then trim the ends of the reads with low sequencing quality (values less than Q20) and remove the reads containing 10% N. Small fragments less than 75 bp in length after removing the adapter and quality trimming were discarded. The Q20 and Q30 values for clean data were 95.84% and 88.33%, respectively, allowing for subsequent analysis. Clean data were spliced using SPAdes v3.14.1 software [19]. Sequences with sufficiently high coverage depth and a long assembly length were selected as candidates and compared to NT libraries to confirm the mitochondrial scaffold sequences, and sequences were concatenated according to overlaps. We used the Burrows-Wheeler-Alignment Tool [20] to map reads to a reference mitogenome of *Holothuria polii* (LR694133.1) to obtain the final *B. argus* mitochondrial genome sequence.

Mitochondrial genes of *B. argus* were annotated using the online MITOS WebServer (http://mitos.bioinf.uni-leipzig.de/index.py, accessed on 3 December 2021) [21] under the invertebrate mitochondrial genetic code, using the default parameters to predict PCGs, tRNA and rRNA genes (accessed on 3 December 2021). The annotation results for rRNA and tRNA were determined using RNAmmer [22] and tRNAscan-SE [23], respectively, and found to be in agreement with the MITOS results. The blank region of the mitochondrial genome was defined as the control region; this was compared with the mitochondrial genome of the reference species to finally determine the control region. The reference mitogenome was used to determine the starting position and orientation of the mitochondrial assembly sequence to obtain the final mitochondrial genome sequence. The start and stop codons of the genes were manually corrected using SnapGene (https://www.snapgene.com/, accessed on 12 December 2021) to obtain a highly accurate set of conserved genes. The sample genome was displayed in a circle using CGView [24]. The relative probability that a particular codon was among the synonymous codons encoding the corresponding amino acid reflected the degree of preference for codon usage [25]. Preference values for codons were obtained by calculating the relative synonymous codon usage (RSCU) using EMBOSS V6.6.0.0 [26]. The bias of the nucleotide composition was calculated using the formulas: AT skew = (A − T)/(A + T); GC skew = (G − C)/(G + C) [27]. CREx was used to compare the gene order in mitochondria and infer the gene rearrangement based on common intervals [28]. Rearrangements included reversals (R), transpositions (T), reverse transpositions (RT) and tandem-duplication-random-losses (TDRL) [29].

### 2.3. Phylogenetic Analysis

In total, the mitochondrial genomes for 32 Holothuroidea, and 4 Echinodermata species (*Echinocardium cordatum*, *Pygmaeocidaris prionigera*, *Crossater pappsus*, *Anthenea aspera*) that served as an outgroup, were obtained from GenBank (Table 1). PhyloSuite was used to extract the nucleotide sequences of 13 PCGs from these mitochondrial genomes [30]. The MAFFT program [31] integrated into PhyloSuite was executed to align multiple sequences in normal-alignment mode with 9 Echinoderm and Flatworm mitochondrial codes, and ambiguously aligned regions were identified and removed by Gblocks [32]. The alignments of individual genes were then concatenated and used to generate input files (Phylip and Nexus formats) for phylogenetic analyses. Phylogenetic trees were built under Bayesian inference (BI) and maximum likelihood (ML) methods. The best-fit model of nucleotide sequences for the BI method was determined as GTR + F + I + G4, and the GTR + F + R4 model was chosen as the best fit for the ML method by ModelFinder [33] according to the Bayesian information criterion. The ML analysis was carried out using IQ-TREE [34] and conducted with 5000 ultrafast bootstrap replicates. The BI analysis was performed in MrBayes 3.2.6 [35] with default parameters and four Markov Chain Monte Carlo generations. The trees were sampled every 2,000,000 generations with a burn-in of 25%. The resultant trees were edited using the ITOL online service (https://itol.embl.de/, accessed on 1 March 2022).

## 3. Results and Discussion

### 3.1. Mitochondrial Genome Structure and Organization

**Figure 1:**
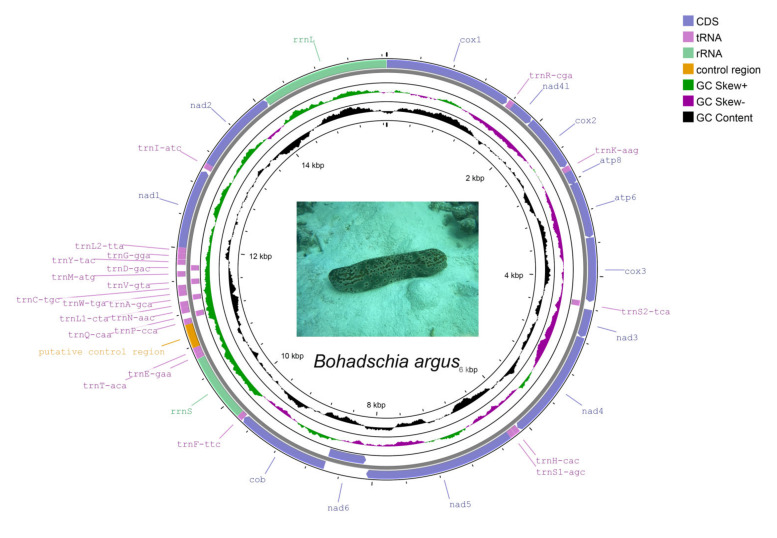
Mitochondrial genome map of *B. argus*. Genes encoded in the forward direction are located on the outside of the ring, while those encoded in the reverse direction are located on the inside of the ring. The black peak represents the deviation of GC%; the deep purple and green peaks represent the deviation of GC skew.

The raw reads were quality filtered, and high-quality clean reads containing 4122.8 Mb were obtained for the assembly of the *B. argus* mitochondrial genome. The whole mitochondrial genome of *B. argus* is a 15,656-bp circular molecule with a nucleotide composition of 26.9% T, 23.35% C, 32.12% A and 17.64% G. The AT content (59.01%) was significantly higher than the GC content (40.99%), indicating that the mitochondrial genome was biased toward A and T bases. In contrast to the AT skew (0.09), the GC skew (−0.14) was negative, which indicated that in the mitochondrial genome of *B. argus*, the A bases occur more frequently than the T bases, and the C bases occur more frequently than the G bases (Table 2). In sea cucumbers, the AT content of the mitochondrial genome varies, but most of the sea cucumbers in the family Cucurbitaceae have a higher AT content than GC content, e.g., *Holothuria polii*, *Holothuria fuscocinerea*, *Holothuria leucospilota* and *Holothuria spinifera* [36,37,38,39]. In bacteria, GC skew is considered a footprint of genome evolution driven by DNA replication [40]. However, little is known about the relationship between the GC skew of the mitochondrial genomes and the species evolution of sea cucumbers. We believe that more mitochondrial genomes of sea cucumbers need to be sequenced to further explore whether GC skew is related to the evolution of sea cucumbers. The mitochondrial genome of *B. argus* encodes 37 genes, including 13 PCGs, two ribosomal RNA genes (*rrnL* and *rrnS*), 22 transfer RNA genes (tRNAs) and a putative control region (Figure 1 and Table 3). *nad6* and 5 tRNA genes (*trnS2*, *trnQ*, *trnA*, *trnV* and *trnD*) were encoded on the light chain (L), and the remaining 31 genes, including 12 PCGs, 17 tRNA genes and 2 rRNA genes, were encoded on the heavy chain (H) (Table 3). In other mitochondrial genomes of sea cucumbers released thus far, all PCGs except *nad6* were encoded on heavy chains. The complete mitochondrial DNA sequence of *B. argus* was deposited in GenBank under the accession number OL741685. Raw sequence data were deposited in the Short Read Archive (SRA) database (https://www.ncbi.nlm.nih.gov/sra/, accessed on 25 April 2022) with the accession no. SRR19025759.

### 3.2. Protein-Coding Genes

The total length of the 13 PCGs is 11,354 bp, accounting for 72.52% of the *B. argus* mitochondrial genome, and the AT and GC contents are 58.9% and 41.1%, respectively (Table 2). The start codon of the 13 PCGs is ATG, which is similar to the eight brittle stars reported by Taekjun Lee [41] and *Benthodytes marianensis* reported by Mu et al. [42]. The stop codon for most PCGs is TAA, with the exceptions being TAG for *nad5* and single T for *cox2* and *nad4* (Table 3). Incomplete termination codons are frequently detected in metazoans, and it is speculated that incomplete stop codons may be formed by posttranscriptional polyadenylation of TAA [43,44,45,46]. 

**Figure 2:**
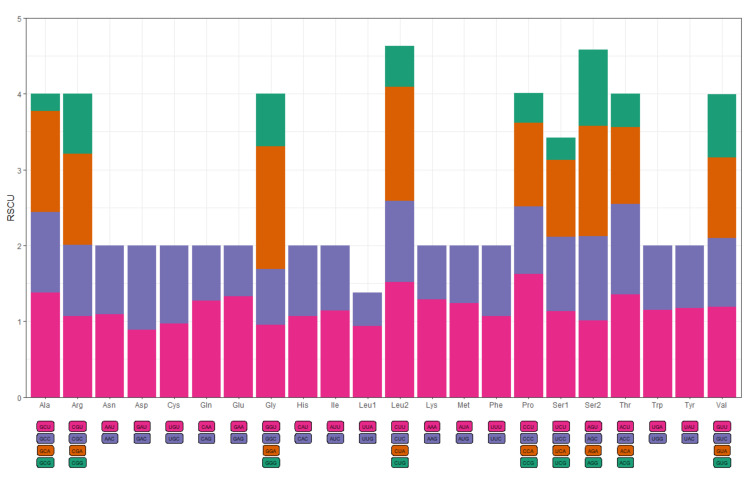
Relative synonymous codon usage (RSCU) in the mitogenome of *B. argus*. The horizontal coordinates represent the amino acids encoded by the codon, and the vertical coordinates represent RSCU values.

The usage of amino acids and RSCU values in the PCGs of *B. argus* are summarized in Table 4 and Figure 2. The most frequently used codons (amino acids) were AAA (Lys), which was used 217 times (4.3%), followed by CCU (Pro) with 146 times (2.9%) and AUA (Met) with 145 times (2.8%). GCG (Ala) was the least used codon, appearing just 12 times (0.2%). Excluding the stop codon, the 13 PCGs of *B. argus* contained a total of 5016 codons. The AT contents of the codons were very similar, with 59.32%, 59.11% and 58.23% when AT were located on the first, second and third position of the codons, respectively. This phenomenon differed from that previously observed in abalone, oyster and scallop, where the AT content at the third codon position was significantly higher than that at the first or the second position in all these species [47,48,49].

### 3.3. tRNAs and rRNAs

**Figure 3:**
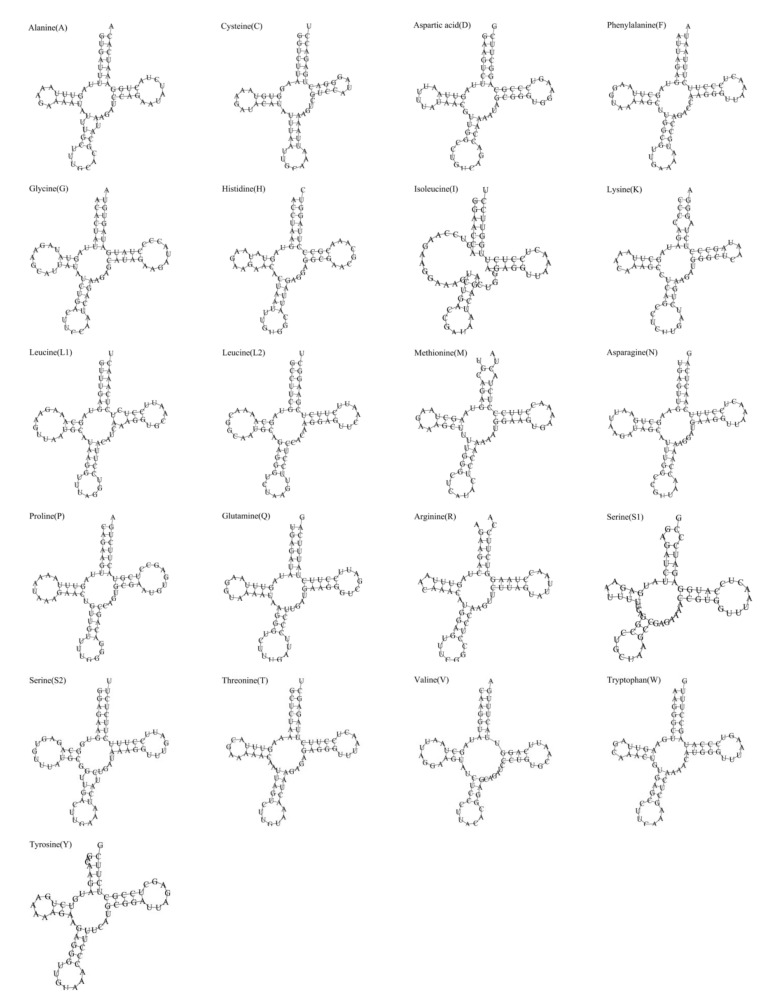
Secondary structure of tRNAs in the mitochondrial genome of *B. argus*.

Similar to other metazoans, the mitochondrial genome of *B. argus* contains 22 tRNA genes. The total length of the 22 tRNA genes is 1525 bp, with tRNA gene lengths ranging from 65 bp (*trnC*, *trnY*) to 73 bp (*trnG*, *trnN*). We examined the secondary structure of 22 tRNAs and found that most had an ordinal cloverleaf structure and that the anticodon arms contained a relatively conserved region (Figure 3). However, simplified loops of dihydrouridine were detected in *trnR*, *trnS1* and *trnW*. Furthermore, the dihydrouridine arm was missing from *trnI*. The deletion of the dihydrouridine arm in *trnI* was also observed in *Holothuria scabra* and *Benthodytes marianensis* [42,50]. It has been suggested that the lack of a dihydrouridine arm or thymidine-pseudouridine-cytidine (TΨC) loop may not affect tRNA function [51,52]. Meanwhile, G-U wobble base pairs were frequently observed to be present in these tRNAs (Figure 3). G-U wobble base pairs are now found in almost every class of functional RNA and have been shown to uniquely play many important chemical and structural roles [53].

The *rrnS* and *rrnL* genes identified in the *B. argus* mitochondrial genome are 830 and 1541 bp in length, respectively. Both genes have a slight GC bias. The *rrnS* gene has the base composition of A = 37.11%, T = 22.05%, C = 20.12% and G = 20.72%, and the *rrnL* gene has the base composition of A = 37.31%, T = 20.05%, C = 22.06% and G =4 2.12%. Both genes are encoded on the H chain, where *rrnL* locates between *cox1* and *nad2* and *rrnS* is near the putative control region between *trnE* and *trnF*.

### 3.4. Control Region and Overlapping Regions

A putative control region with a total length of 290 bp was predicted between *trnT* and *trnP* in the *B. argus* mitochondrial genome. The AT content (60.69%) and GC content (39.31%) of this region were similar to those of the whole mitochondrial genome. Both AT skew (−0.26) and GC skew (−0.16) were negative, which indicated that the A content was lower than the T content and that the G content was lower than the C content (Table 2). Most metazoans have only one control region in the mitochondrial genome [54], and a few have two [42,55,56]. A total of six overlapping regions were detected, ranging from 1 to 7 bp in length; the longest was 7 bp located between *atp8* and *atp6*, and the shortest was 1 bp between *trnF* and *cob*, *trnA* and *trnL1*, and *trnV* and *trnC*.

### 3.5. Gene Arrangement

**Figure 4:**
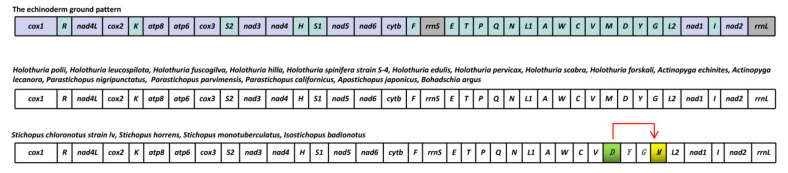
Linearized representation of the conserved mitochondrial gene order in 20 holothuroid species. A red arrow represents a gene arrangement event of tandem-duplication-random-losses (TDRL).

Shared gene arrangements may imply common ancestry as the same gene order is unlikely to occur independently in separate lineages [57]. Gene arrangement comparisons may be a reliable tool for studying phylogeny when some ancestral relationships are focused [58]. The 20 complete mitochondrial genome sequences in Aspidochirotida were used to compare the gene orders in mitochondrial genomes. The results revealed that 19 species, including *B. argus*, shared gene blocks in the ground pattern of echinoderms, while *Stichopus chloronotus* strain *lv*, *Stichopus horrens*, *Stichopus monotuberculatus* and *Isostichopus badionotus* showed a different gene arrangement event of tandem-duplication-random-losses (TDRL) (Figure 4). *tRNA-Met* moved downstream of *tRNA-Gly*, which is consistent with a previous report [59].

### 3.6. Phylogenetic Analysis

**Figure 5:**
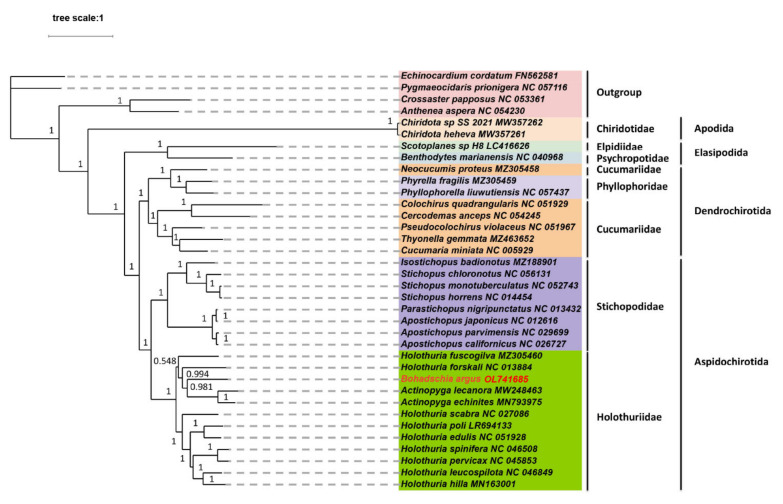
Phylogenetic tree derived from Bayesian inference (BI) of 13 mitochondrial PCGs in *B. argus* and other closely related species. Bayesian posterior probabilities are shown next to corresponding nodes.

We determined the complete mitochondrial genome of *B. argus* to further investigate the phylogeny of Holothuroidea. The results indicated that the BI and ML analyses yielded phylogenetic trees with highly similar topologies, but the tree based on BI had higher support values (Figure 5 and Figure S1). This indicated that all the orders in Holothuroidea are highly monophyletic (BI posterior probability (PP) = 1; ML bootstrap (BP) = 100), with the exception being Cucumariidae. Holothuriidae and Stichopodidae formed a sister group.

Both Chiridotida and Elasipodida belong to the deep-sea allopatric branch, and they clustered together to form two deep-sea branches. Previous reports showed that during the Late Devonian, the differentiation between deep-sea and neritic sea cucumbers was located at 386.93 Mya [29]. 

Five species from Holothuriidae and seven species from Stichopodidae formed a sister group and were classified as Aspidochirotida. This classification was supported by our morphological observations. However, in previous phylogenetic studies, Stichopodidae clustered first with the Phyllophoridae and Cucumariidae of Dendrochirotida and then with the Holothuriidae when using 16S rRNA genes as target sequences [60]. In the phylogenetic tree constructed based on COI genes, Holothuriidae, Cucumariidae and Phyllophoridae were clustered into one group, and two genera of Stichopodidae were clustered into another group [60]. The phylogenetic trees constructed based on the two genes reflected that Stichopodidae and Holothuriidae, which belong to the same Aspidochirotida, are more distantly related [60]. The results suggested that discrepant phylogenetic results are more likely occur when using single genes. Lacey et al. used the 18S rRNA gene to analyze the phylogenetic relationships among dozens of species of sea cucumbers, including Stichopodidae, Holothuriidae, Cucumariidae and Phyllophoridae, and the results showed that Stichopodidae did not cluster with Holothuriidae either [61]. This may be since the 18SrRNA gene is a nuclear gene. As this demonstrates, with the growing prevalence of high-throughput sequencing, phylogenetic analyses based on all PCGs in complete mitochondrial genomes can provide us with a more reliable understanding of evolutionary relationships among sea cucumbers.

We also found that *Actinopyga lecanora* and *Actinopyga echinites* were clustered into a branch with a high nodal support value (PP = 1; BP = 100). This is consistent with the result of the phylogenetic tree constructed using 13 PCGs by Zhong et al. [14]. *B. argus* clustered into one branch with the clade of *A. lecanora* and *A. echinites*, which revealed that the *B. argus* and *Actinopyga* species are relatively closely related. Interestingly, *B. argus*, *A. lecanora* and *A. echinites* have an important defense organ, the cuvierian organ, which is released when they feel danger or make contact with stimuli [62]. That same defense mechanism suggests that they may have shared similar evolutionary mechanisms. Pearson (1914) considered that two genera, *Actinopyga* and *Bohadschia*, were closely related when compared with *Holothuria* [63]. However, in an evolutionary analysis using 16SrRNA genes, Keer et al. found that Actinopyga and Bohadschia were each monophyletic with high bootstrap percentages [64]. The specific evolutionary relationship between Actinopyga and Bohadschia requires further exploration. Additionally, *H. forskali* and the clade formed by *B. argus*, *A. lecanora* and *A. echinites* were clustered into one branch. In previous studies, researchers also revealed that *Actinopyga* and *H. forskali* are relatively closely related using whole mitochondrial genomes [65]. 

It is worth noting that the Holothuriidae species were not clustered by generic relationships, which we may surmise given how the PCGs sequences for Bohadschia, Actinopyga and Holothuria were interwoven. These interlaced clades suggest that the evolutionary relationships between species of different genera in Holothuriidae have not been elucidated, possibly due to the lack of mitochondrial genomic data. In the future, a greater quantity of mitochondrial genomic data will be necessary to further elucidate the evolutionary relationships of sea cucumbers in Holothuriidae. 

## 4. Conclusions

In this study, the mitochondrial genome of *B. argus* was acquired using next-generation sequencing technology. Thirteen PCGs, 22 tRNAs, two rRNAs, and a 290-bp putative control region were annotated. Comparative genomics revealed that the *B. argus* mitochondrial genome has typical gene contents, and the gene order is consistent with the ancestral arrangement of echinoderms. The results of the phylogenetic analysis supported the proposal that *Bohadschia* belongs as a member of Holothuriidae and is closely related to species in *Actinopyga* and *Holothuria*. Holothuriidae and Stichopodidae formed a sister group, and they were both clustered into Aspidochirotida. These results fill a gap in the mitochondrial genomes of *Bohadschia* and will contribute to improving our understanding of the evolution of *Bohadschia*.

## Figures and Tables

**Table 1:** Mitogenomes of sea cucumbers used for the phylogenetic analysis in this study.

Family	Species	Sizes (bp)	Accession No.
Holothuriidae	*Holothuria polii*	15,907	LR694133.1
Holothuriidae	*Holothuria leucospilota*	15,904	NC_046849.1
Holothuriidae	*Holothuria fuscogilva*	15,633	MZ305460.1
Holothuriidae	*Holothuria hilla*	15,744	MN163001.1
Holothuriidae	*Holothuria spinifera strain S-4*	15,812	NC_046508.1
Holothuriidae	*Holothuria edulis*	15,743	NC_051928.1
Holothuriidae	*Holothuria fuscocinerea*	15,633	MZ305460.1
Holothuriidae	*Holothuria scabra*	15,779	NC_027086.1
Holothuriidae	*Holothuria forskali*	15,841	NC_013884.1
Holothuriidae	*Actinopyga echinites*	15,619	MN793975.1
Holothuriidae	*Actinopyga lecanora*	15,568	MW248463.1
Stichopodidae	*Stichopus monotuberculatus*	16,274	NC_052743.1
Stichopodidae	*Stichopus chloronotus strain lv*	16,247	NC_056131.1
Stichopodidae	*Stichopus horrens*	16,257	NC_014454.1
Stichopodidae	*Parastichopus nigripunctatus*	16,112	NC_013432.1
Stichopodidae	*Isostichopus badionotus*	16,318	MZ188901.1
Stichopodidae	*Parastichopus parvimensis*	16,120	NC_029699.1
Stichopodidae	*Parastichopus californicus*	16,727	NC_026727.1
Stichopodidae	*Apostichopus japonicus*	16,099	NC_012616.1
Psychropotidae	*Benthodytes*	17,567	NC_040968.1
Elpidiidae	*Scotoplanes*	15,910	LC416626.1
Phyllophoridae	*Phyllophorella liuwutiensis*	15,969	NC_057437.1
Phyllophoridae	*Phyrella fragilis*	15,910	MZ305459.1
Cucumariidae	*Thyonella gemmata voucher UF:021831*	15,696	MZ463652.1
Cucumariidae	*Neocucumis proteus*	16,495	MZ305458.1
Cucumariidae	*Cercodemas anceps*	16,539	NC_054245.1
Cucumariidae	*Pseudocolochirus violaceus*	15,756	NC_051967
Cucumariidae	*Colochirus quadrangularis*	17,157	NC_051929.1
Cucumariidae	*Cucumaria miniata*	17,538	NC_005929.1
Chiridotidae	*Chiridota sp. SS-2021*	17,199	MW357262.1
Chiridotidae	*Chiridota heheva*	17,200	MW357261.1

**Table 2:** Nucleotide composition and AT-GC skewness of the *B. argus* mitogenome.

*B. argus*	Size	A%	T%	G%	C%	AT%	GC%	AT-Skew	GC-Skew
mitogenome	15,656	32.12	26.9	17.64	23.35	59.01	40.99	0.09	−0.14
PCGs	11,354	29.97	28.93	17.08	24.02	58.90	41.10	0.02	−0.17
tRNAs	1525	31.02	27.8	22.23	18.95	58.82	41.18	0.05	0.08
rRNAs	2371	37.24	21.09	20.29	21.38	58.33	41.67	0.28	−0.03
control region	290	22.41	38.28	16.55	22.76	60.69	39.31	−0.26	−0.16

**Table 3:** Characteristic features of the mitochondrial genome of *B. argus*.

Gene	Direction	Location	Size	Start Codon	Stop Codon	Anticodon
*cox1*	+	1–1554	1554	ATG	TAG	
*trnR*	+	1564–1630	67			CGA
*nad4l*	+	1631–1927	297	ATG	TAA	
*cox2*	+	1929–2616	688	ATG	T	
*trnK*	+	2617–2682	66			AAG
*atp8*	+	2683–2850	168	ATG	TAA	
*atp6*	+	2844–3527	684	ATG	TAA	
*cox3*	+	3530–4312	783	ATG	TAA	
*trnS2*	−	4312–4382	71			TCA
*nad3*	+	4405–4749	345	ATG	TAA	
*nad4*	+	4753–6109	1357	ATG	T	
*trnH*	+	6111–6178	68			CAC
*trnS1*	+	6180–6247	68			AGC
*nad5*	+	6248–8080	1833	ATG	TAG	
*nad6*	−	8097–8585	489	ATG	TAA	
*cob*	+	8594–9736	1143	ATG	TAA	
*trnF*	+	9736–9806	71			TTC
*rrnS*	+	9808–10,637	830			
*trnE*	+	10,640–10,709	70			GAA
*trnT*	+	10,710–10,779	70			ACA
*trnP*	+	11,070–11,141	72			CCA
*trnQ*	−	11,138–11,207	70			CAA
*trnN*	+	11,209–11,281	73			AAC
*trnL1*	+	11,282–11,353	72			CTA
*trnA*	−	11,353–11,420	68			GCA
*trnW*	+	11,421–11,488	68			TGA
*trnC*	+	11,489–11,553	65			TGC
*trnV*	−	11,553–11,622	70			GTA
*trnM*	+	11,653–11,721	69			ATG
*trnD*	−	11,727–11,796	70			GAC
*trnY*	+	11,797–11,861	65			TAC
*trnG*	+	11,864–11,936	73			GGA
*trnL2*	+	11,937–12,007	71			TTA
*nad1*	+	12,008–12,979	972	ATG	TAA	
*trnI*	+	13,003–13070	68			ATC
*nad2*	+	13071–14,111	1041	ATG	TAA	
*rrnL*	+	14,112–15,652	1541			
Controlregion		10,780–11,069	290			

**Table 4:** Codon numbers and relative synonymous codon usage in the *B. argus* mitochondrial genome.

Codon	Count	RSCU	Codon	Count	RSCU
UUU(F)	137	1.07	UCU(S)	103	1.13
UUC(F)	119	0.93	UCC(S)	89	0.98
UUA(L)	84	0.94	UCA(S)	93	1.02
UUG(L)	39	0.44	UCG(S)	26	0.29
CUU(L)	136	1.52	CCU(P)	146	1.62
CUC(L)	96	1.07	CCC(P)	80	0.89
CUA(L)	134	1.50	CCA(P)	100	1.11
CUG(L)	48	0.54	CCG(P)	35	0.39
UAU(Y)	119	1.17	AUU(I)	108	1.14
UAC(Y)	84	0.83	AUC(I)	81	0.86
UAA(*)	125	1.24	AUA(M)	145	1.24
UAG(*)	77	0.76	AUG(M)	88	0.76
CAU(H)	78	1.07	GUU(V)	56	1.19
CAC(H)	68	0.93	GUC(V)	43	0.91
CAA(Q)	94	1.27	GUA(V)	50	1.06
CAG(Q)	54	0.73	GUG(V)	39	0.83
ACU(T)	117	1.35	AAU(N)	140	1.09
ACC(T)	104	1.20	AAC(N)	118	0.91
ACA(T)	87	1.01	AAA(K)	217	1.29
ACG(T)	38	0.44	AAG(K)	119	0.71
GCU(A)	72	1.38	GAU(D)	60	0.89
GCC(A)	55	1.06	GAC(D)	75	1.11
GCA(A)	69	1.33	GAA(E)	114	1.33
GCG(A)	12	0.23	GAG(E)	58	0.67
UGU(C)	43	0.97	CGU(R)	34	1.07
UGC(C)	46	1.03	CGC(R)	30	0.94
UGA(W)	74	1.15	CGA(R)	38	1.20
UGG(W)	55	0.85	CGG(R)	25	0.79
AGU(S)	92	1.01	GGU(G)	54	0.95
AGC(S)	101	1.11	GGC(G)	42	0.74
AGA(S)	133	1.46	GGA(G)	92	1.62
AGG(S)	91	1.00	GGG(G)	39	0.69

* indicates termination codon.

## Data Availability

The genome sequence data that support the findings of this study are openly available in the NCBI GenBank at https://www.ncbi.nlm.nih.gov/ (accessed on 7 March 2022) under accession no. OL741685.

## Supplementary Materials

The following supporting information can be downloaded at: https://www.mdpi.com/article/10.3390/ani12111437/s1, Figure S1: Phylogenetic tree inferred from the nucleotide sequences of 13 mitogenome protein-coding genes using the maximum likelihood (ML) method. Numbers on branches indicate bootstrap.
