# Supplementary material for: Analysis of Complete Mitochondrial Genome of Bohadschia argus (Jaeger, 1833) (Aspidochirotida, Holothuriidae)
Source: Animals (Basel). 2022 Jun 2;12(11):1437. doi: 10.3390/ani12111437 (PMC9179316; doi:10.3390/ani12111437)
Supplement: Supplementary file 1 [file animals-12-01437-s001.zip › animals-1721811-supplementary.pdf]

animals

Supplementary Materials

Analysis of Complete Mitochondrial Genome of *Bohadschia argus* (Jaeger, 1833) (Aspidochirotida, Holothuriidae)

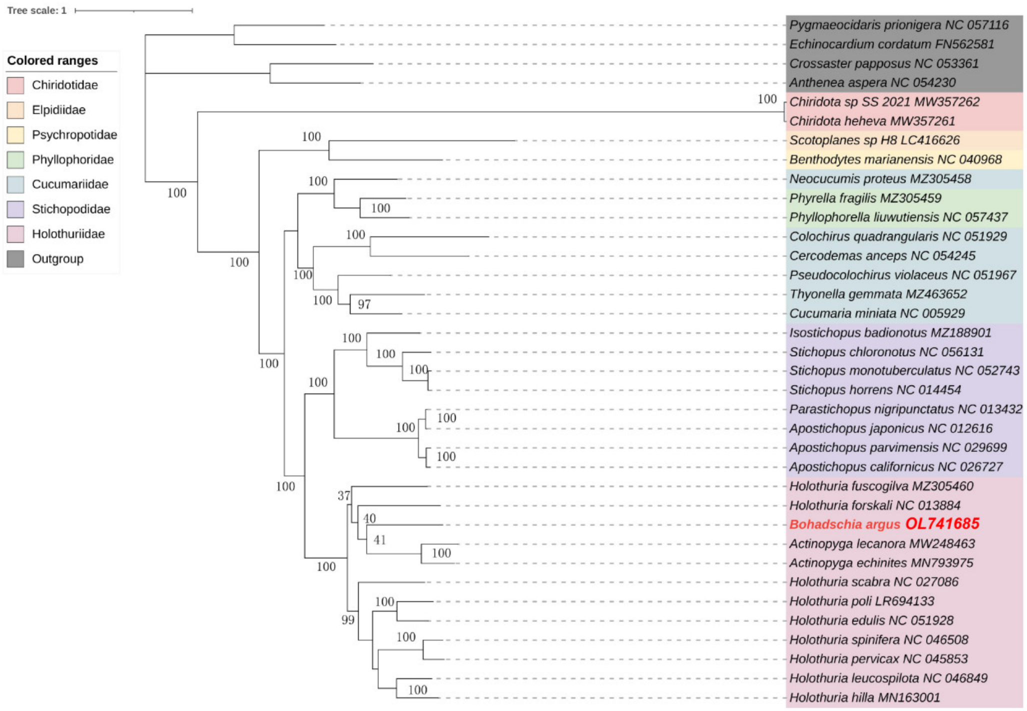

Figure S1. Phylogenetic tree inferred from the nucleotide sequences of 13 mitogenome protein-coding genes using the maximum likelihood (ML) method. Numbers on branches indicate bootstrap.
